# Enhanced thermal effect using magnetic nano-particles during high-intensity focused ultrasound

**DOI:** 10.1371/journal.pone.0175093

**Published:** 2017-04-06

**Authors:** Surendra Balaji Devarakonda, Matthew R. Myers, Dushyanth Giridhar, Seyed Ahmad Reza Dibaji, Rupak Kumar Banerjee

**Affiliations:** 1 Department of Mechanical, Materials Engineering, College of Engineering and Applied Science, University of Cincinnati, Cincinnati, Ohio, United States of America; 2 Division of Solid and Fluid Mechanics, Center for Devices and Radiological Health, U.S. Food and Drug Administration, Silver Spring, Maryland, United States of America; Ohio State University, UNITED STATES

## Abstract

Collateral damage and long sonication times occurring during high-intensity focused ultrasound (HIFU) ablation procedures limit clinical advancement. In this reserarch, we investigated whether the use of magnetic nano-particles (mNPs) can reduce the power required to ablate tissue or, for the same power, reduce the duration of the procedure. Tissue-mimicking phantoms containing embedded thermocouples and physiologically acceptable concentrations (0%, 0.0047%, and 0.047%) of mNPs were sonicated at acoustic powers of 5.2 W, 9.2 W, and 14.5 W, for 30 seconds. Lesion volumes were determined for the phantoms with and without mNPs. It was found that with the 0.047% mNP concentration, the power required to obtain a lesion volume of 13 mm^3^ can be halved, and the time required to achieve a 21 mm^3^ lesion decreased by a factor of 5. We conclude that mNPs have the potential to reduce damage to healthy tissue, and reduce the procedure time, during tumor ablation using HIFU.

## Introduction

High intensity focused ultrasound (HIFU) is a noninvasive modality that is seeing growing application for the treatment of solid tumors and metastatic disease. In typical HIFU ablation procedures, the temperature in a tissue volume roughly 1 mm in radius is raised above 60°C, resulting in essentially instantaneous cell destruction via coagulative necrosis. HIFU has been applied to treat solid tumors in the pancreas, liver, prostate, breast, and uterus. In comparison to conventional tumor/cancer treatment methods, such as open surgery, radio- and chemo-therapy, HIFU has the advantages of non-invasion, non-ionization, and fewer complications after treatment [1].

While HIFU possesses multiple technical advantages, several challenges also exist that limit its clinical application. Skin burns arising from long treatment times can occur [2]. Unablated tumor cells in spaces between small focal regions have also been observed [3], as has undesired tissue damage at higher acoustic powers [4]. To overcome these obstacles, the ability to use lower acoustic powers and/or the ability to necrose with shorter duration times would be advantageous. Microbubble contrast agents have been used to achieve the same lesion volume by using lower acoustic powers and shorter durations [4]. Exogenous absorbers such as nanoparticles can also be applied to the tumor tissue, which allows more precise and intense heating of the tumor site, reducing damage to healthy tissue [5]. Nanoparticle systems are also well suited for drug delivery to the tumors, as intravenous drug delivery can lead to undesired bio-distribution and unwanted accumulation in healthy tissues [6, 7]. In addition, nanoparticles offer the opportunity to develop multifunctional platforms for integrated imaging and therapy [8].

Sun et al.[9, 10] reported the effects of PLGA-coated Fe_3_O_4_ microcapsules in HIFU therapy and have found enhancement in hyperthermia due to the microcapsules. However, the authors pointed out that the size of the microcapsules used by them were relatively large (500–850 nm). Use of such large size particles are non-physiological and may be difficult to translate to clinical studies. In addition, the acoustic powers used for ablation in these studies was significantly high (180–250 W). Such higher powers are known to cause skin burns and damage to neighboring healthy cells[5]. Also, the MR images of the HIFU thermometry reported by the authors lack clarity in quantifying the temperature rise within the tumor region. The unclear image could be due to the higher particle concentration (3.1 mg/ml) used in the *in-vivo* studies resulting in decreased signal to noise ratio. The magnetism of mNPs are reported to interfere with MR imaging as discussed by Etheridge et al.[11].

Dibaji et al. [12] measured the HIFU-induced temperature rise using embedded thermocouples (TCs) in tissue phantoms with different (0%, 1%, and 3%) concentrations of magnetic nanoparticles (mNPs). They determined that the peak temperature rise increased by 1.6 and 2 times when mNPs concentration of 1% and 3% were used, respectively, for an acoustic power of 14.2 W. However, the temperature rise was measured by focusing on the TC junctions, which induced artifacts [13–15]. Additionally, the mNPs concentrations used were toxic and considerably above those used in clinical practice[16].

In the present study, the effect of physiologically acceptable concentrations of mNPs on the HIFU-induced temperature rise, thermal dose, and lesion volume has been assessed. Tissue phantoms with 0% (control), 0.0047%, and 0.047% mNPs concentrations by volume were fabricated. The concentrations of mNPs used in this study were more than 20 times lower than those used previously by Dibaji et al.[12], hence, are considered to be below the toxicity range (31 mg/ml to 58.5 mg/g)[16]. Each tissue phantom was embedded with four TCs and sonication was performed in the center of the TC array using transducer acoustic powers of 5.2 W, 9.2 W, and 14.5 W. The temperature profiles during the heating and cooling periods were recorded at each embedded TC.

Thus, in this study, the following main outcomes were evaluated,

- An inverse algorithm [17] was used to estimate the *temperature rise* at the focus and other radial locations.
- The transient focal temperature profiles were used for *thermal dose* calculation. The temperature rises and the thermal doses were compared for different mNP concentrations and acoustic powers.
- *Lesion volumes* were calculated and compared for different concentrations of mNPs.
- The *sonication time* required to achieve necrosis (as defined by a threshold thermal dose) was computed for cases with and without mNPs.

According to our knowledge database, quantification of NPs mediated thermal therapy endpoints (discussed above) using focused acoustic energy (high intensity focused ultrasound [HIFU]) has not been reported elsewhere.

## Methods

### Fabrication of tissue phantoms

**Fig 1:**
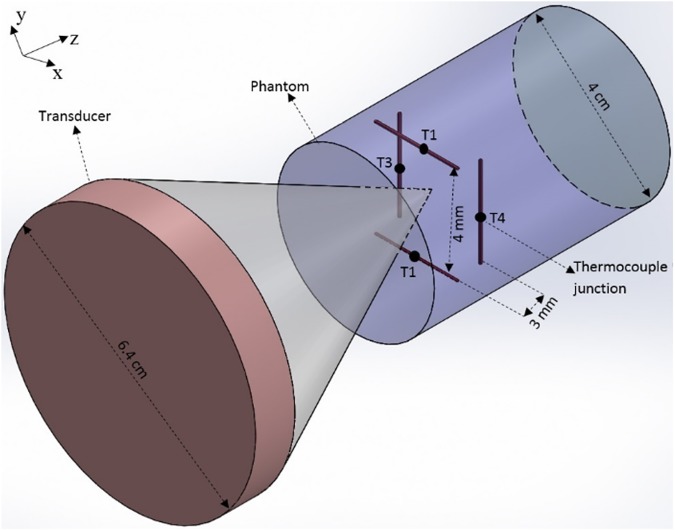
Schematic of the transducer and the phantom with four TCs.

Four cylindrical fixtures with a length of 5 cm and inner diameter of 3 cm (volume = 35.34 cm^3^) were developed. Each fixture was embedded with an array of four thin-wire (Chromega-Constantine) TCs (labeled T1-T4) with the diameter of 0.003 in, arranged in two layers (Fig 1). According to the TC-accuracy chart provided by the manufacturer, the standard limit of error for the TCs was the greater of 1.7°C or 0.5% of the measured temperature. Each layer had two TCs with wires that were parallel to each other, and the TC wires in one layer were oriented perpendicular to those in the other layer. The two TCs in each layer were separated by a distance of 4 mm, and each layer was 3 mm in axial extent away from the adjacent layer (Fig 1). The bottom of the cylindrical fixtures were closed using a glass plate.

A 35.34 mL gelrite-based TMM was prepared according to the protocol of King et al. [18]. To prepare a tissue phantom with 0% mNPs (control) concentration by volume, the 35.34 mL liquid TMM was poured into the first fixture (volume = 35.34 cm^3^) until the fixture was filled. The construction of phantoms with mNPs was explained in detail in Dibaji et al. [12]. Using this method, phantoms with 0.0047% and 0.047% mNPs concentration were fabricated. During the fabrication of the phantom, mNPs were uniformly dispersed inside the TMM by mixing them thoroughly, avoiding aggregations of mNPs [11]. All the tissue phantoms were kept at room temperature for about 12 hours to ensure complete solidification of the poured liquid.

### Sonication procedure

**Fig 2:**
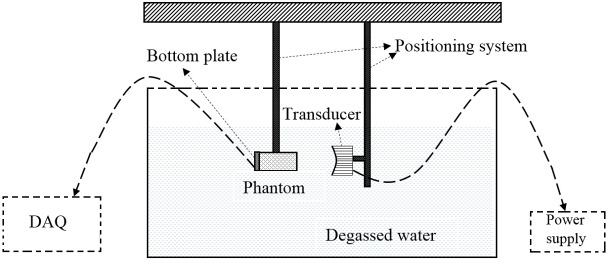
Experimental setup showing the transducer, phantom and the DAQ.

Fig 2 shows the experimental setup used for performing HIFU sonications. An H102 transducer (Sonic Concepts Inc., Bothell, WA) with a focal length of 6.26 cm, outer diameter of 6.4 cm, and inner diameter of 2.2 cm was used as the source of the ultrasound. The transducer was driven in continuous-wave mode by a signal generator (33220A, Agilent Technologies) with an operating frequency of 1.025 MHz. A 150-Watt amplifier (150A100B, Amplifier Research) was used to amplify the signal. Both the transducer and the phantom were kept submerged inside degassed water during the experiments. The transducer was attached to a positioning system which was capable of adjusting any of the coordinates (x, y, and z) in discrete 0.025 mm increments.

The following sonication-procedure was performed for all of the phantoms. The beam was positioned in the center of the TC array (Fig 1) in such a way that all the four TC junctions recorded approximately the same temperature rise for a sonication period of 10 sec. After positioning the beam in the center of the array, the transducer was activated in a continuous wave mode for a period of 30 sec. The temperature on the full array, i.e., T1, T2, T3, and T4, was recorded using an OMB-DAQ-56 (Omega Engg. Inc., Stamford, CT) data acquisition system over the 30 sec heating period, and 20 sec cooling period (total time = 50 sec). The temporal resolution of the temperature measurements was 0.5 sec. Three transducer acoustic powers of 5.2 W, 9.2 W, and 14.5 W were used for the sonication. These powers correspond to values at which the transducer was previously calibrated using a radiation-force balance. Three trials (*n* = 3) were performed for each power level. After each temperature recording, the tissue phantoms were allowed to cool down to the ambient water temperature (24°C).

### Micro-Computed Tomography (Micro-CT) imaging

**Fig 3:**
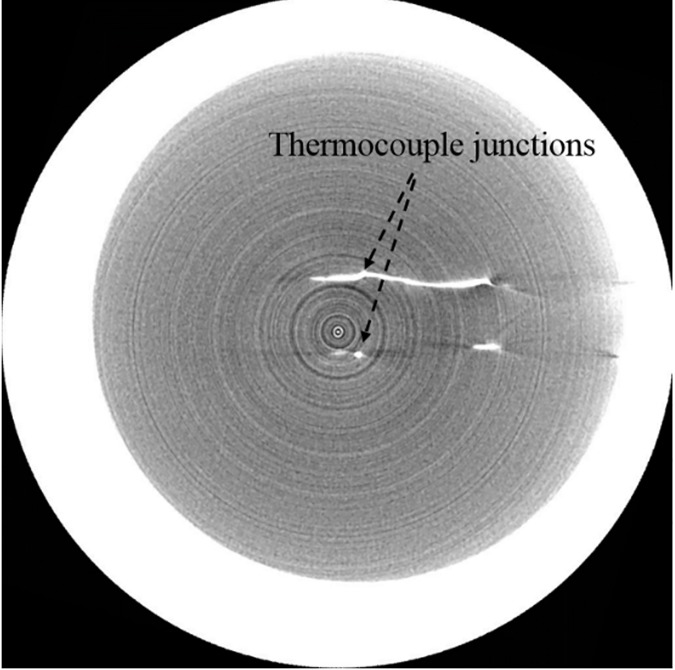
CT image of TCs within phantom. The bright spots represent the TC junctions.

During pouring of the TMM into the fixture, TC wires could be displaced slightly from their original position. As a result, high resolution micro-CT (Inveon Multimodality System, Siemens Inc., Germany) was used to scan the tissue phantoms, in order precisely determine the location of the TC junctions, which are later used in the inverse algorithm [17]. The processing software used in conjunction with the CT scan images was Inveon Research Workplace 4.2. The acquisition software used for obtaining the CT images was Inveon Acquisition Workplace (IAW) 2.0.2. The micro-CT images obtained with a voxel size of 36 μm were brighter for tissue phantoms with higher mNPs concentration compared to the control phantom. Fig 3 shows the TC junctions as bright spots inside the phantom. The position of these junctions were determined for the control phantom and the phantoms containing 0.0047% and 0.047% mNPs. The TC junction locations were later used in determining the focal location of the beam.

### Beam localization within TC array

As a first step in determining the focal temperature, the beam location within the TC array was ascertained, using an inverse algorithm published by Hariharan et al. [17]. The inverse method was used because it significantly reduces TC artifacts. As reported by Morris et al. [13], temperature rise due to TC artifact can exceed temperature rise due to ultrasound absorption in the medium of interest. In the inverse algorithm, the coordinates of the beam focus, relative to the TC coordinates just obtained from the CT scans, are treated as unknowns. The location of the beam is adjusted until the difference between the computed temperature rise at the array nodes and experimentally measured values at those locations is minimized. The beam-focus is located by the coordinates yielding the minimum error.

### Focal temperature

Once the beam location was determined, it was used in a solution to the heat equation in the form of an exponential-integral [17, 19] to determine the temperature at locations and times of interest. Dillon et al.[19] assumed a heat source that was constant in the axial direction and possessed a Gaussian profile in the radial direction. The approximation is useful when the radial variation in heat is much stronger than the axial one, and the observation point is located close to the beam axis (less than the axial beamwidth). Under these conditions, the temperature distribution as a function of time at a distance r from the beam axis can be estimated from:

T(r,t)=αI0r022κρ0cp[Ei(−r2r02)−Ei(−r2r02(1+4κtr02)] (1A)

where Ei(*x*) is the exponential integral:

$$Ei(x)=-\int_{-x}^{\infty }\frac{e^{-s}}{s}ds$$ (1B)

Here *r* is the radial coordinate, *r*_0_ is the beam radius with of the Gaussian intensity distribution, *α* is the absorption coefficient, which is a function of nanoparticle concentration, *I*_0_ is the intensity on the beam axis, *κ* is the thermal diffusivity, *ρ*_0_ is the density, and *c*_*p*_ is the specific heat of TMM. The properties of the TMM are presented in Table 1. The exponential integral was used for a variety of applications, as described below.

**Table 1:** Properties of the TMM.

Property	Value
**Density, *ρ*_0_**	1040 kg/m^3^
**Absorption Coefficient, α**	45 dB/m
**Specific Heat, *C***_***p***_	4064 J/kg.K
**Thermal Diffusivity, *κ***	1.4 x 10^−7^ m^2^/s

### Thermal dose

The thermal dose corresponding to a temperature *T(t)* was calculated using the method developed by Sapareto and Dewey [20]. The thermal dose parameter is given by

$$t_{43}(x,y,z)=\int_{t=0}^{t=t_{final}}R^{43-T(t)}dt$$ (2)

where *t*_43_ is the thermal dose at the reference temperature of 43°C, *t*_*final*_ is the treatment (sonication + cooling) time, *T*(*t*) is the temperature (in °C) as a function of time obtained experimentally at the focal location, and

$$R=\left\{\begin{array}{l} \;0.5\;if\;T(t)\geq 43{}^{\circ}C\\ 0.25\;otherwise \end{array}\right\}$$ (3)

For all thermal-dose calculations, a trapezoidal scheme was used to perform the integration with a time increment of *dt* = 0.5 sec.

### Lesion volume

A lesion was defined as a volume of cells exposed to a threshold thermal dose [21]. In principle, the threshold appropriate for the particular organ and species of interest should be used. As these values are often not available, a value of 240 equivalent min [21] is often used, and was used in our calculations.

**Fig 4:**
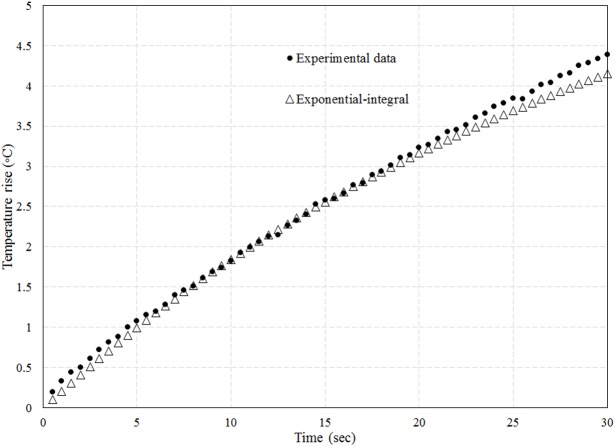
Experimental temperature trace at TC T1 along with fitting curve based upon exponential-integral formulation. Power = 5.2 W and mNP concentration = 0.047%.

**Fig 5:**
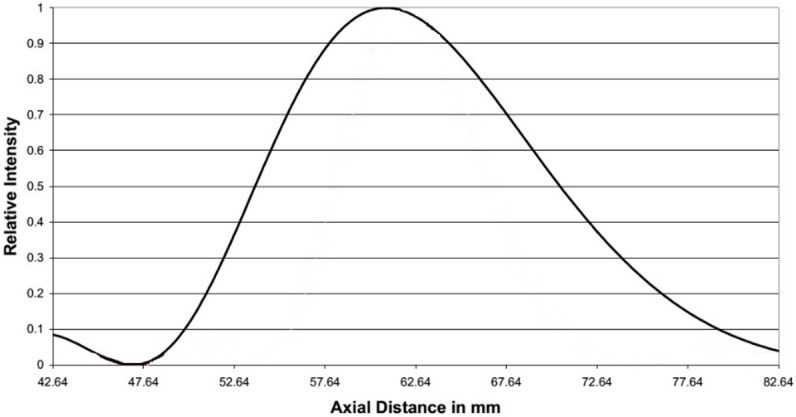
Relative intensity of the transducer along the axis for the fundamental frequency.

Lesion volume was computed through repeated use of the exponential integral (Eq 1), to compute the temperature at locations where it was not measured. Eq 1 requires knowledge of the on-axis absorbed energy α*I*_*0*_ and radius *r*_*0*_ of the ultrasound beam for a given set of exposure conditions (e.g. mNP concentration) and location of interest. The parameters α*I*_*0*_ and *r*_*0*_ in the T1-T2 plane were found by varying their values and choosing the pair that resulted in a temperature trace (defined by Eq (1)) that most closely matched that at the location of T1 (or T2, since the two traces were similar, the beam being equidistant from both). Varying the values for α*I*_*0*_ and *r*_*0*_ consisted of generating a grid of 11 x 11 values for the pair (α*I*_*0*_ and *r*_*0*_), each choice resulting in a change in peak temperature of about 0.5 deg C. The pair resulting in the closest root-mean-square (r.m.s.) agreement with the experimental trace was selected, provided the r.m.s. error was less than 3%. Fig 4 shows the temperature predicted by the exponential integral function alongside the experimental values, for fitting parameters of α*I*_*0*_ = 0.14 x 10^−3^ W/mm^3^ and *r*_*0*_ = 1.17 mm. The mNP concentration for Fig 4 is 0.047% and the power is 5.2 W. The quantity α*I*_*0*_ outside the T1-T2 plane could be obtained from the relative intensity profile for the transducer, assuming the absorption coefficient to be the same at the two locations. The profile for the fundamental frequency (obtained from the manufacturer, Sonic Concepts Inc., Bothell WA.) is shown in Fig 5. The beam width at locations outside the T1-T2 plane was computed using a conservation-of-energy relation: the product of axial intensity and the square of the beam radius is constant, except for the attenuation loss between the two axial locations.

**Fig 6:**
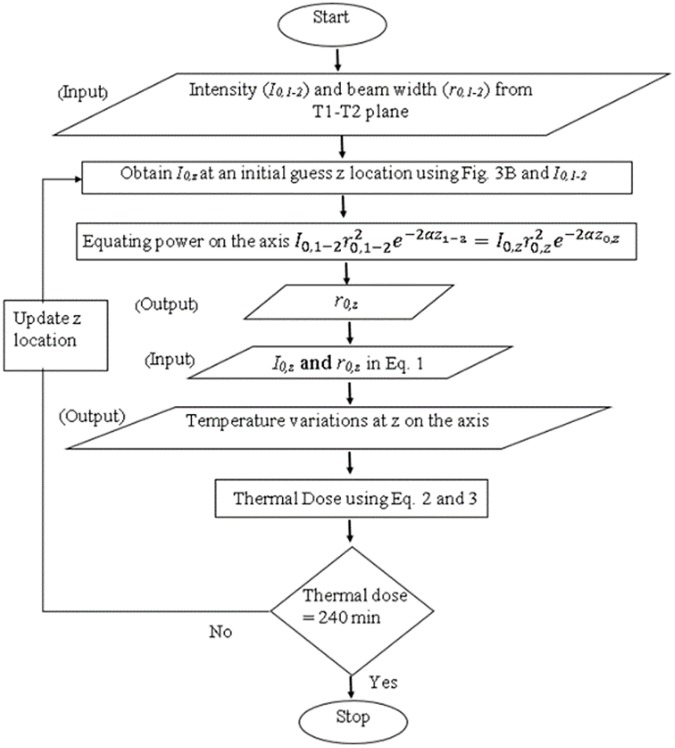
Flow chart to determine the z location with thermal dose of 240 equivalent minutes.

To quantify the lesion volume, the locations along the z and r axes where the thermal dose was equal to a minimum of 240 equivalent minutes were calculated for the end-of-sonication time of interest. Fig 6 presents the flow diagram of the steps involved in determining the axial extent of the lesion. In the algorithm, an initial guess *z* was made for the axial boundary of the lesion. At that axial location, the on-axis absorbed intensity α*I*_*0*_*(z)* relative to the value in the T1-T2 plane was determined from the axial intensity profile (Fig 5) for the transducer, as described above. The beam radius *r*_*0*_ was then determined from the conservation of energy relation. The temperature profile in the plane at axial location z was subsequently found from Eqs (1A) and (1B). The temperature was then used in Eq (2) to determine the thermal dose in the plane at location z. If the thermal dose was not within 3% of 240 equivalent minutes, the guess for z was updated and the procedure repeated. The radial coordinate of the 240-minute point was found using a similar iterative process.

**Fig 7:**
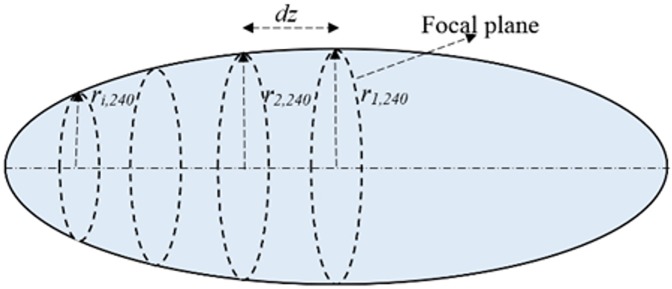
Discretization of lesion volume into truncated cones.

Lesion volume was computed by considering each section of axial length *dz* (Fig 7) to be a truncated cone of height *dz*, and base radii r^i^_240_ and r_i+1,240_ for the i^th^ section. The lesion volume (V) was calculated by summing up the volumes of the truncated cones:

$$V=\sum \frac{1}{3}\pi (r_{i,240}^{2}+r_{i,240}r_{i+1,240}+r_{i+1,240}^{2})dz$$ (4)

The increment *dz*, taken to be 4 mm initially, was halved until continued halving resulted in a change in lesion volume of less than 5%. The final increment was *dz* = 0.1 mm.

### Absorbed energy

In addition to temperature rise, thermal dose, and lesion volume, another useful metric of the enhanced heating due to the presence of mNP’s is the absorbed ultrasound energy. This can be computed from the initial slope ∂*T*/∂*t* of the temperature trace. For sufficiently small times, heat conduction can be neglected and the temperature rise is related to the absorbed energy Q by a simplified form of the heat equation:

$$\rho \;c_{p}\frac{\partial T}{\partial t}=Q=2\;\alpha \;I$$ (5)

The ratio of the absorbed ultrasound energy with and without mNP’s was computed for the different powers considered.

### Extrapolation using the exponential integral

The exponential-integral formulation (1) was used to extrapolate the experimental results for cases where the power or duration time was not sufficiently high to create a lesion (thermal dose did not exceed 240 equivalent minutes), to conditions where a lesion volume could be computed. The extrapolation to higher powers proceeded by first computing the absorbed energy *αI*_*0*_ on axis, using the experimental data and exponential integral as described above (and illustrated in Fig 4), for all the combinations of transducer powers and mNP concentrations where lesions were not created. A line was then fit through the absorbed energy vs transducer power results, in order to extrapolate to higher powers. The beam width was found to be independent of power, and hence no extrapolation was performed. Once the extrapolation line for higher absorbed energies was available, higher values of the absorbed energy *αI*_*0*_ could be considered in the calculations, and referenced back to extrapolated higher transducer powers.

Increasing the thermal dose by lengthening the exposure duration could be easily done by increasing the time in Eq (1), once the parameters *αI*_*0*_ and *r*_*0*_ were determined for any given set of conditions.

### Uncertainty in temperature estimates

In a previous study by the authors[17], it was determined that the uncertainty in temperature estimates performed using the exponential-integral technique was approximately 6% in the focal plane. The uncertainty arises due to inexact knowledge of the TC location, variation in thermal properties, and limitations on the assumption of source that is invariant in the axial direction and Gaussian in the radial direction. Away from the focal plane the accuracy decreases due to beam curvature; 6 mm below the focal plane the error in the temperature measurements was found to be about 20%[17]. To determine the total uncertainty in the temperature estimates in this study, a 6% uncertainty was added to the standard deviation of temperature for the three trials.

## Results

The HIFU-induced temperature rises were measured in a tissue phantom with the three acoustic powers (5.2 W, 9.2 W, and 14.5 W) and three mNP concentrations (0%, 0.0047%, and 0.047%). The temperatures of the degassed water and the pre-sonication tissue phantoms were 24°C. The measured temperatures were averaged over three trials for each acoustic power level. Results are presented as mean ± SD.

**Fig 8:**
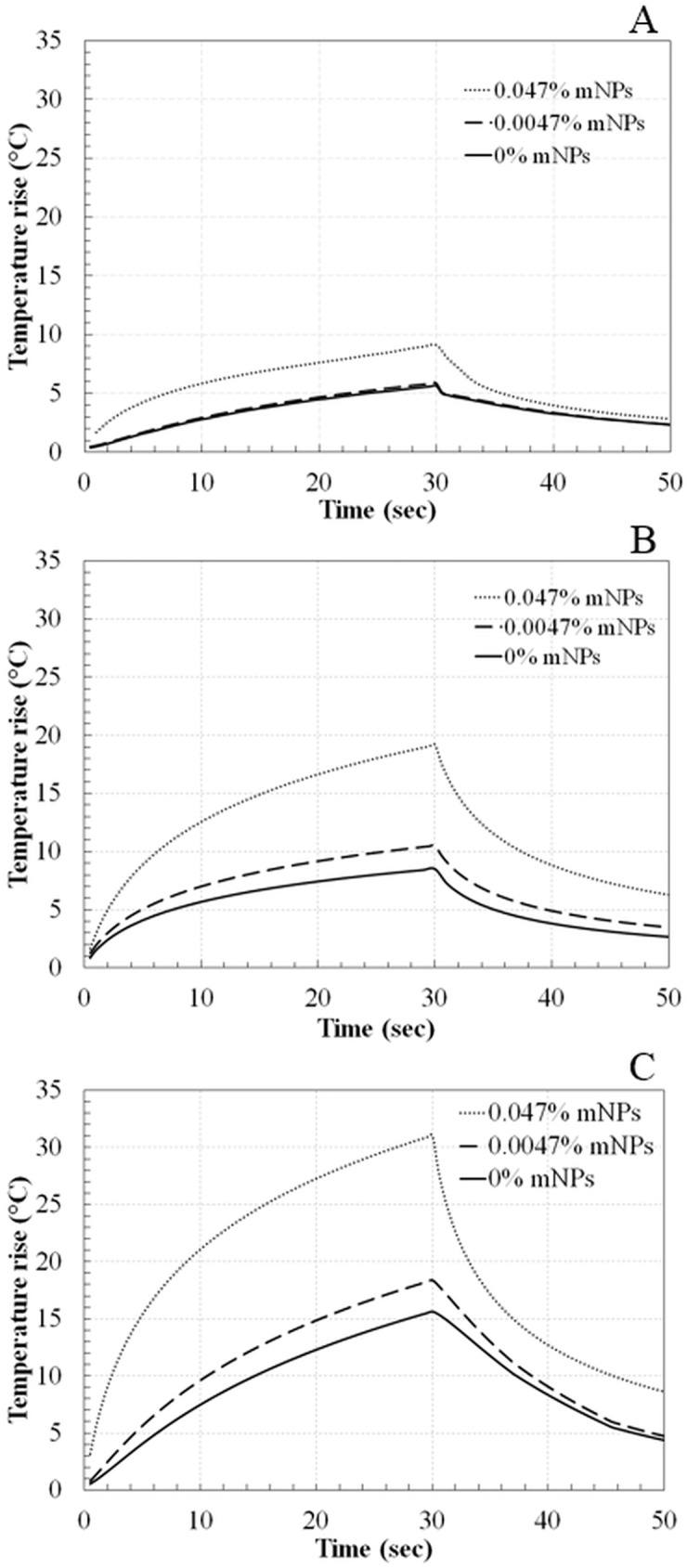
Temperature variation with time at focus in tissue phantoms with 0%, 0.0047%, and 0.047% mNPs concentrations using acoustic power of (A) 5.2 W, (B) 9.2 W, and (C) 14.5 W for a sonication of 30 sec.

**Fig 9:**
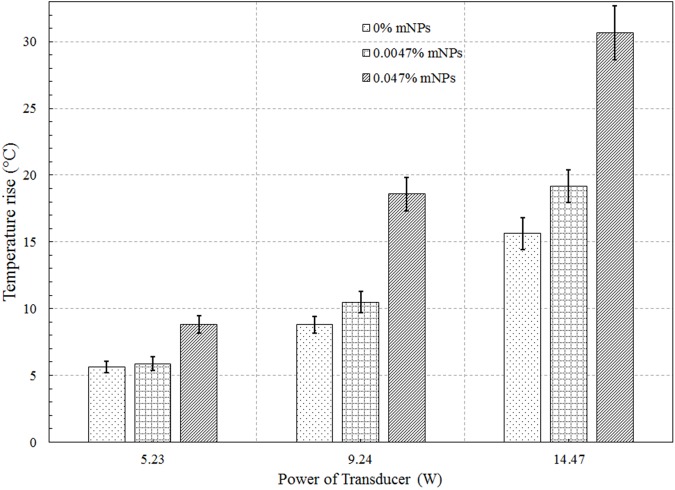
Temperature rise (at end of sonication) at the focus for 5.2, 9.2, and 14.5 W acoustic powers.

Fig 8 shows the HIFU-induced focal temperature variations with time for the three mNP concentrations and three power levels. The focal temperatures were derived using Eq (1), according to the procedure of the previous section. The end-of-sonication temperatures are plotted in Fig 9. At the two higher powers, the end-of-sonication temperature for the 0.047% mNP concentration is approximately double that for the control case (0% mNP). The temperature rise for the 0.0047% concentration is within 20% of the temperature rise for the control phantom, at all powers (S1 File).

The amount of absorbed ultrasound energy, derived from the initial slope of the temperature trace, is provided in Table 2. Results are normalized by the absorbed energy in the absence of mNP’s. An initial time of 2.5 sec was chosen to compute the initial slopes. The 2.5 sec time was chosen because it was long enough that the temperature rise due to ultrasound attenuation elevated above the TC noise level on the remote TCs, yet short enough that appreciable diffusion did not yet occur [12]. For the power of 5.2 W, approximately 1.1 times more energy was absorbed with the 0.0047% mNP concentration than with no mNP’s, and 2.9 times more energy relative to control for the 0.047% concentration. For the 9.2 W power level, the relative energy absorption was 1.2 for the 0.0047% mNP concentration and 1.4 for the 0.047% mNP concentration. Finally, for 14.5 W, the relative energy absorption was 1.5 for the 0.0047% mNP concentration and 4.6 for the 0.047% mNP concentration.

**Table 2:** The ratio of the initial slope of the temperature trace, normalized by the slope for the case of 0% mNPs at each selected power.

	5.2 W	9.2 W	14.5 W
**0.0047% mNPs**	1.1	1.2	1.5
**0.047% mNPs**	2.9	1.4	4.6

**Fig 10:**
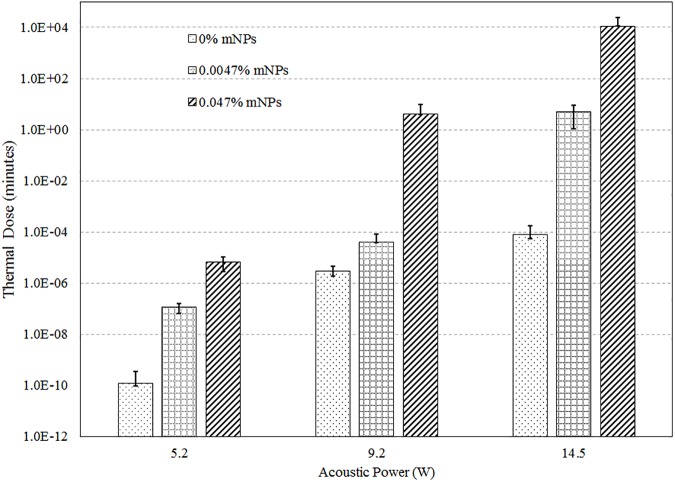
Thermal dose at the focus for 5.2, 9.2, and 14.5 W acoustic powers and 0%, 0.0047%, and 0.047% concentrations of mNPs (Y-axis is in log scale).

The thermal dose at the beam focus was computed using Eqs (2 and 3). The computed thermal doses are plotted on a logarithmic scale in Fig 10. The thermal dose at the beam focus increased between 1 and 4 orders of magnitude relative to no mNP’s for the 0.0047% mNP level and 5 to 8 orders of magnitude for the 0.047% mNP level (S2 File).

The sonication time required to produce a lesion 5 mm in axial extent and 1 mm radius (21 mm^3^ volume) is reported in Table 3. The extrapolation technique discussed in the previous section was used to extend heating times beyond the 30 seconds used in the experiments. At the highest power level, the time required to produce the lesion decreases by a factor of 2 with the addition on 0.0047% mNP’s, and a factor of 5 with 0.047% concentration mNP’s.

**Table 3:** The estimated sonication time required to obtain a lesion volume of 21 mm^3^ (r = 1 mm and z = 5mm) for phantoms with three different mNP concentrations (0%, 0.0047%, and 0.047%), for acoustic powers of 5.2 W, 9.2 W, and 14.5 W.

	5.2 W	9.2W	14.5 W
**0% mNPs**	71000 ± 9000 sec	2200 ± 300 sec	166 ± 21 sec
**0.0047% mNPs**	59000 ± 7700 sec	770 ± 100 sec	82 ± 10 sec
**0.047% mNPs**	2200 ± 300 sec	92 ± 13 sec	31 ± 4 sec

**Fig 11:**
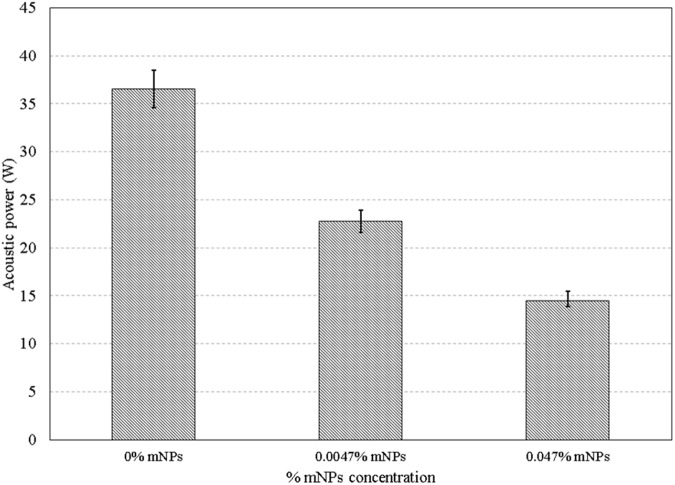
Acoustic powers required to obtain a lesion volume of 13.5 mm^3^ for 0%, 0.0047%, and 0.047% mNPs at 1.025 MHz for a sonication period of 30 sec.

The acoustic power required to achieve a lesion volume equal to 13 mm^3^—the volume achieved at the highest power level (14.5 W) and highest mNP concentration (0.047%)—was calculated for the two lower mNP concentrations (0%, 0.0047%). These powers, determined using the extrapolation method described in the previous section, are plotted in Fig 11. The 14.5 Watts required at the 0.047% mNP concentration increased to 22.8 Watts for a 0.0047% concentration, and to 36.6 W for no nanoparticles (S3 File).

## Discussion

The remote TC method employed in this study eliminated the uncertainties associated with positioning the beam atop a TC. A tradeoff for this decrease in uncertainty is an uncertainty associated with the determination of the TC locations. The TMM was poured into the fixtures without directly contacting TCs and care was taken to construct all phantoms in the same manner. Additionally, the CT scan helped reduce the uncertainty in the TC locations. The uncertainty varied from 0.3°C for 0% mNPs and 5.2 W to 1.8°C for 0.047% and 14.5 W.

While data on only 1 TC (e.g. T1 or T2) over time was necessary for providing the input into the exponential integral (Eq 1), the four TCs were used to increase the accuracy of the localization method. As determined by Hariharan et al. [17], the error in the prediction of the focal location using 4 TCs is approximately 0.2 mm, about 20% of the error using 2 TCs and 40% of the error for 3 TCs. Using more than 4 TCs increases the accuracy only marginally.

The exponential-integral (Eqs 1A and 1B) is a solution to the heat equation that ignores axial diffusion. The error associated with the approximation of purely radial diffusion can be estimated by considered the relative importance of axial and radial diffusion in the heat equation. If the relevant length scale in the radial direction is *l*_*r*_, and the length scale in the axial direction is *l*_*z*_, then the importance of axial diffusion relative to radial diffusion is roughly *(l*_*r*_ */l*_*z*_*)*^*2*^. Estimates of the radial and axial length scales are the radial and axial beamwidths. For the transducer used in this study operated in the fundamental mode, the ½ -power axial beam width is approximately 17 mm (Fig 5). The radial beam width, obtained from a similar, radial profile, is 1.4 mm. The error in ignoring axial diffusion is roughly (1.4/17)^2^, or about 0.6%.

The acoustic energy absorption, as quantified by the initial slope of the temperature trace, increased substantially (up to a factor of almost 5) with the addition of mNP’s. For the 0.047% concentration and a power of 9.2 W, the absorption was lower than trends under other, similar conditions would dictate. This low value is possibly associated with the difficulty in computing slopes from numerical data.

The HIFU absorption in media embedded with mNPs depend on the thermal processes within the *viscous* and *phonon layers* at the interface of mNPs as well as on the *intrinsic absorption* properties of the media [22–25]. Propagating HIFU waves in a medium interact with the thermal phonons and consequently, a part of the wave is absorbed. Due to the wave absorption, there is an increase in the momentum of thermal phonons inside the medium leading to temperature rise. It is expected that the attenuation due to phonon layer is the dominating mechanism for the mNPs size of 10 nm leading to temperature rise, but further analysis is required. Such analysis may need an independent theoretical-experimental characterization study that we have embarked on.

**Fig 12:**
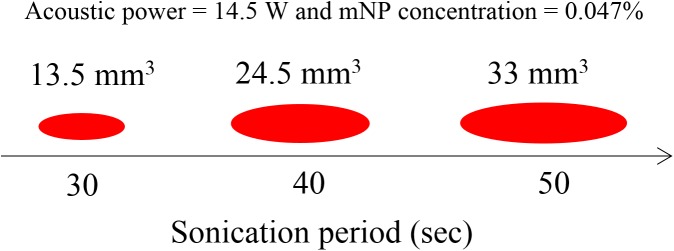
Lesion volume for a sonication period of 30, 40, and 50 sec for a mNP concentration of 0.047% and acoustic power of 14.5 W.

The increased acoustic attenuation caused by the presence of nanoparticles led to a substantial increase in the temperature rise and the thermal dose (Figs 9 & 10) at the focal region inside the phantoms. The time to produce a 21 mm^3^ lesion decreased by about a factor of 5 with the addition of mNP’s in the 0.047% concentration. This represents a substantial time savings for clinical ablation procedures. Viewed another way, the power required to produce such a lesion in 30 seconds decreased by a factor of more than 2 (Fig 11), thereby reducing the risk of collateral damage to healthy tissue. Lesion volumes have been plotted by extrapolating the experimental data for the mNPs concentration of 0.047% and 14.5 W. The lesion volume increased by 1.8 and 2.4 times when the sonication time was increased to 40 and 50 sec, respectively, when compared to a sonication period of 30 sec (Fig 12).

The extrapolated times required for lesion formation (Table 3) should be viewed as rough estimates, as accuracy of the predictions derived from Eq 1 cannot be ensured for times that are orders of magnitude higher than those used to calibrate the model. Still, the computed times illustrate that the presence of the mNP’s has a substantial influence on the completion times for HIFU ablation procedures.

Toward the goal of determining a dose of mNP’s that is useful from a clinical perspective, a concentration of 0.047% produces substantial effects, e.g. a reduction in sonication time by a factor 5. If biocompatibility or other issues call for a lower mNP dose, a concentration of 0.0047% still produces clinically relevant effects, e.g. a reduction by a factor of 2 in sonication time.

Using the enhanced permeability and retention (EPR) effect [26, 27] wherein the nanoparticles have the tendency to accumulate in the tumor tissue, nanoparticles can be intravenously injected and made to collect in the tumor tissue [28] and obtain tumor-specific targeting [29]. These deep-seated deposits can then be excited by HIFU and treated non-invasively. To develop this technique, future *in-vitro* experiments should be performed using nanoparticles injected into the region of interest with a syringe. Thermal analyses similar to those of the present study can then be performed to evaluate the salutary influence of the mNP’s.

## Supporting information

S1 File Temp Peak. Comparison of peak temperatures for different mNP concentrations and acoustic powers.
(XLSX)

S2 File Thermal Dose. Comparison of thermal dose for different mNP concentrations and acoustic powers.
(XLSX)

S3 File Power comparison. Acoustic powers required to obtain a lesion volume of 13.5 mm^3^ for different mNPs concentration.
(XLSX)
